# Case Report of a Hemangioblastoma With Large Blood Vessels and Rare Vascular Anomalies: Is It Fibromuscular Dysplasia or Arteriovenous Malformation Association?

**DOI:** 10.7759/cureus.24527

**Published:** 2022-04-27

**Authors:** Luis A Rodríguez-Hernández, Marcos V Sangrador-Deitos, Humberto Montano-Tello, Michel Mondragon-Soto, Martha Lilia L Tena Suck

**Affiliations:** 1 Neurosurgery, Instituto Nacional de Neurología y Neurocirugía, Mexico City, MEX; 2 Neurology, Instituto Nacional de Neurología y Neurocirugía, Mexico City, MEX; 3 Neurological Surgery, Instituto Nacional de Neurología y Neurocirugía, Mexico City, MEX; 4 Neuropathology, Instituto Nacional de Neurología y Neurocirugia, Mexico City, MEX

**Keywords:** degenerative pathologies, fibromuscular dysplasia, arteriovascular, malformation, fibromuscular dysplasia

## Abstract

Hemangioblastoma is considered a benign neoplasm characterized by abnormal vasculature and stromal cells; several pathophysiological mechanisms have been proposed, such as genetic predisposition, hormonal factors, and arterial wall ischemia. Fibromuscular dysplasia is characterized by hyperplasia or thinning of the smooth muscle, elastic fibre destruction, fibrous tissue proliferation, and arterial wall disorganization. We present a cerebellar hemangioblastoma case not associated with Von Hippel Lindau syndrome. Histologically we evidenced big vessels with anomalies of the vascular walls corresponding to fibromuscular dysplasia, and those changes have not been described in these types of tumors. In this light, rare findings could be called vascular malformations or degenerative vascular changes, fibromuscular dysplasia or vascular anomalies. Arterio-venous malformation and hemangioblastoma pathology are rarely presented together. Notwithstanding, we could say that it is a stromal stem cell tumor in a varied stage of differentiation.

## Introduction

Hemangioblastoma (HB) is characterized by stromal neoplastic cells and abundant blood vessels. It is an uncommon and highly vascular tumor usually observed in adults that are located in the brain stem, cerebellum, spinal cord and retina, and corresponds histologically to grade 1 neoplasms [1]. Cellular and reticular variants are distinguished by abundant stromal components. They represent 1% to 2% of all brain tumors [1]. It may be solid or cystic and approximately 25% to 40% are associated with von Hippel-Lindau syndrome (VHL) [1]. Clinical manifestations of HB are nonspecific and they depend on location and growth pattern. On immunohistochemistry, it usually expresses inhibin A, glucose transporter 1 (GLUT1), CD34, CD31, factor VIII and vimentin, also early growth response (EGR), vascular endothelial growth factor receptor 1 (VEGFR-1), basic fibroblast growth factor (βFGF) as well as platelet-derived growth factor (PDGF) in the vessels. Stroma cells are positive for neuron-specific enolase (NSE), neural cell adhesion molecule (NCAM), vimentin, aquaporin 1, D2-40, CD10, brachyury, hypoxia-inducible factor-1 alpha (HIF-1a), and glial fibrillary acidic protein (GFAP), while being negative for cytokeratin and epithelial membrane antigen (EMA) [1]. 

## Case presentation

**Figure 1:**
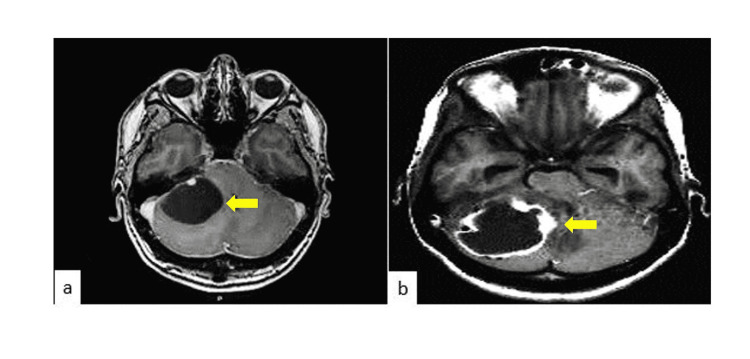
Axial view of the MRI of the brain a: T1 weighted image (T1W), b: T2 weighted image (T2W) T1W, T2W and FLAIR images show a well-defined cystic lesion at the left cerebellum hemisphere of the brain. The T1W axial and T2W coronal images show a well-defined cystic lesion from the dorsally located mural nodule.

A 39-year-old female with a familiar history of unspecified brain tumor was evaluated with a seven-month history of progressive weakness, difficulty in swallowing and staggering gait. On physical examination, gait lateralization, an acute visual deficit of the right eye, left nystagmus, left dysmetria and dysdiadokokinesia were noted. An MRI showed a hypointense on T1 weighted image (T1W) images, and a hyperintense on T2 weighted image (T2W), a mass lesion, occupying the left cerebellar hemisphere associated with a cystic component and obstructive hydrocephalus suggesting a cerebellar HB (Figure 1).

**Figure 2:**
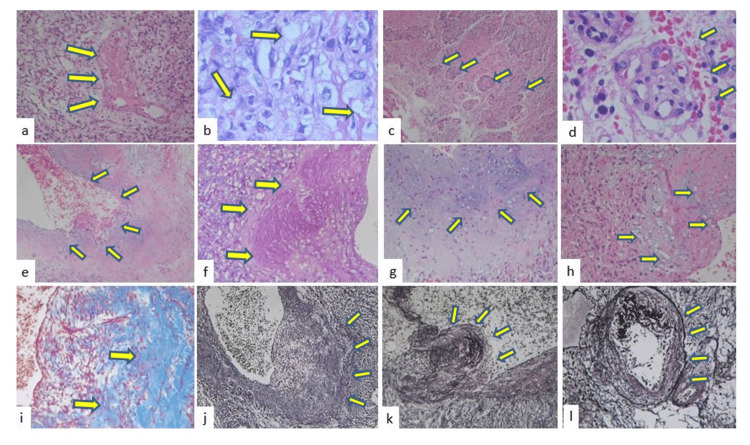
Histopathological analysis A proliferation of blood vessels lined with an endothelium without atypia, intermingled with solid areas consisting of vacuolated stromal cells is seen. a: Small and fine vessels interspersed with stromal cells and fibrinoid necrosis are observed; b: Some stromal cells with the appearance of clear cells; c: Fibrovascular hyperplasia; d: Proliferation of endothelial cells; e: The big vessels show fibrovascular hyperplasia; f: Periodic acid–Schiff (PAS) stain shows this fibrovascular hyperplasia (x400); g: Myxoid changes, tissue appearance similar to chondroma; h: A proliferation of small vessels vascular lumen and stromal cells are observed in the large hyaline areas; i: Dense blue hyaline stroma in Masson's staining (x400); j: Reticulum staining shows distorted and fragmented wall vessels (x200); k: Vessels with dysplastic changes such as sclerosis areas, l: Irregularities of the wall with fragmented fibres are observed (reticulin stain x400)

**Figure 3:**
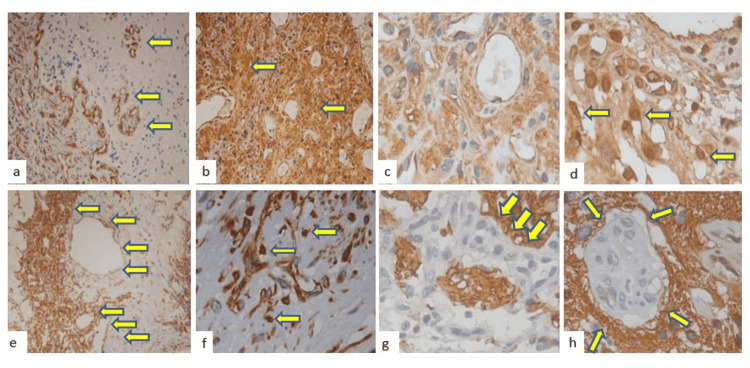
Immunohistochemistry a: The fine vessels are positive for CD34; b: The stromal cells are positive for hypoxia-inducible factor 1-alpha (HIF-1α); c): Vascular endothelial growth factor receptor 2 (VEGFR2) immunoexpressing x200; d: Glucose transporter 1 (GLUT1) stromal cells expression; d: Alfa-1-chymotrypsin nuclear immunoexpressing (x400); e: Vimentin is positive on the wall of the small vessels and in some endothelial cells (x200); f: Vimentin positive reaction in the wall vessels and cells, and vascular lumens is observed; g-h: Glial fibrillary acidic protein (GFAP) stroma positive immunoexpression around the neoplastic cells (original magnification x40).

The gross aspect of the tumor corresponded to several irregular fragments of tissue measuring 30x30mm. They were reddish-brown, highly vascular, with a hemorrhagic appearance. Histology and immunohistochemistry revealed a classic HB (Figure 2, 3).

## Discussion

Hemangioblastoma is a tumor characterized by stromal cells and small vessels [1]. The stromal cells represent the stem cells which, under the neoplastic influence, proliferate and differentiate into “vasoformative elements” creating new blood vessels. However, other types of vascular anomalies are little known in HB. Especially, in large vessels, that mimic arterio-venous malformation [2], or myofibrotic malformation vessels [3]. Medvedev et al. [3] proposed a common embryologic origin as a possible explanation for arteriovenous malformations (AVMs) and HB coexistence. Also, fibro-muscular-dysplasia (FMD) has been reported [4]. Fibro-muscular-dysplasia is a noninflammatory, nonatherosclerotic vasculopathy that usually affects the internal carotid and vertebral arteries and renal arteries characterized by concentric/eccentric smooth muscle collarette around the tunica media of the artery and the vein; also, a variety of degrees of intimal and medial hyperplasia and adventitial fibrosis is presented [5]. The etiology of FMD is not well understood. Several mechanisms have been suspected, such as genetic predisposition and arterial wall ischemia [5]. A large proportion of stromal cells in some tumors express smooth muscle proteins and are potential pericyte precursors [6]. Dysfunction of the von Hippel-Lindau syndrome (VHL) protein causes the accumulation and activation of HIF which can be established in the earliest tumorigenesis stages and is an expression of VEGF, erythropoietin, nitric oxide synthase and GLUT1 in VHL-deficient tumor cells [6]. Differential diagnoses considered within this review are: the vascular anomalies (VAs) divided into tumors and malformations that are distinguished histologically by endothelial cell proliferation, such as lobular capillary hemangiomas and tufted angiomas that are complaints of aberrant angiogenesis [6]. Recent research supports that both pathologies; HB and AVM have an embryologic origin but require later genetic alterations [7]. 

With blood vessels, differences between the lesion mentioned in the present case and the so-called "feeding vessels" described in hemangioblastoma and other neoplasms outside the central nervous system (CNS) are not clear. In the case of hemangioblastoma being a highly vascularized tumor, it is possible to identify blood vessels of greater calibre with irregular and thickened walls at the expense of proliferation of the intimal tunic, myxoid degeneration and secondary loss of the internal elastic lamina. As long as the biopsy corresponds to a peripheral area of ​​the lesion associated with the mural nodule, the lesion could be differentiated from its initial angioglioma description (which corresponds to glioma as a low-grade tumor) with a prominent vascular component that resembles cavernous angioma, arteriovenous malformation or hemangioblastoma. In 1914, Councillmann et al. [8] coined the term “angioglioma” to describe a cerebellar tumor with a large vascular component, histologically characterized by dilated venous channels embedded in a densely fibrillary glial neoplasm with variably sized venous channels along the wall of the cyst. Remarkably in 2013, Yano et al. [9] reported 74 cases of brain tumors associated with AVM, considering these as a whole multidisciplinary lesion mass.

## Conclusions

As of today, it is difficult to conclude the coexistence of AVM and HB within the same pathology. Hemangioblastoma shows rare vascular changes, as vessels with disorganized, thickened walls and abnormal proliferation. Consideration for a differential vascular diagnosis suggests hemangioma, fibromuscular dysplasia or arterio-venous malformation. The present case would be the first one reported of hemangioblastoma with histologically dysplastic changes reported. Further research is needed to elucidate the rare intermixture origin of these lesions.
